# Genome and drug resistance analysis of *Mycobacterium abscessus* complex on tropical islands in China

**DOI:** 10.3389/fmicb.2026.1702466

**Published:** 2026-02-04

**Authors:** Jieying Wang, Chunrong Li, Xianliang Zheng, Zhuolin Chen, Wen Ye, Yuni Xu, Wenhua Qiu, Shaowen Chen, Hua Pei, Yeteng Zhong

**Affiliations:** 1 Department of Clinical Laboratory, The Second Affiliated Hospital of Hainan Medical University, Haikou, China; 2 Department of Clinical Laboratory, Guangxi Medical University Cancer Hospital, Nanning, Guangxi, China; 3 Department of Clinical Laboratory, People’s Hospital of Changshan, Quzhou, Zhejiang, China

**Keywords:** *Mycobacterium abscessus* complex (MABC), average nucleotide identity (ANI), pan-genome, virulence gene, drug resistance

## Abstract

**Objective:**

Based on whole-genome sequencing (WGS) technology, the species distribution, genetic correlations, virulence and drug resistance gene characteristics of the clinical isolates of *Mycobacterium abscessus* complex (MABC) in the tropical island of China (Hainan) were analyzed to provide a basis for clinical precise diagnosis and treatment.

**Methods:**

A total of 113 MABC strains from the Second Affiliated Hospital of Hainan Medical University from 2014 to 2023 were collected. Whole-genome sequencing (WGS) was used for subspecies identification (Average Nucleotide Identity, ANI), genetic distance analysis (single nucleotide polymorphism, SNP), and pan-genome analysis. The distribution of virulence and antibiotic resistance genes was analyzed through the Virulence Factor Database (VFDB) and the Comprehensive Antibiotic Resistance Database (CARD). The drug susceptibility phenotypes were detected by the microbroth dilution method.

**Results:**

Among the 113 MABC strains, *M. abscessus* subsp. *abscessus* (Mab) accounted for 65.5%, *M. abscessus* subsp. *massiliense* (Mma) accounted for 33.6%, and *M. abscessus* subsp. *bolletii* (Mbo) accounted for 0.9%. The strains showed high genetic diversity among them, but two pairs of Mab strains with high genetic similarity (differing by 5 and 8 SNPs respectively) were identified, suggesting the possibility of local common exposure. The pan-genome is open-ended and consists of 3,626 core genes and highly variable accessory/unique genes. The virulence genes show species-specific differences: the detection rate of the phenolic lipid synthesis gene *papA5* in Mma is significantly higher than that in Mab (92.1% vs. 47.3%, *p* < 0.001), while the PDIM synthesis gene *ppsE* is significantly lower in Mma (0.0% vs. 98.6%, *p* < 0.001). The drug sensitivity test shows that the 14-day induction of resistance rate of Mma to clarithromycin is significantly lower than that of Mab (5.3% vs. 86.5%, *p* < 0.001). Resistance genes are widely carried, including *rrs* a1408g (99.1%), *rrl* a2059g (98.2%) mutations, and the efflux pump gene *qacJ* in Mma (97.4%).

**Conclusion:**

In Hainan region, environmental exposure is the main source of MABC infection. Whole genome analysis suggests the potential risk of local common exposure. Mma has a better treatment prospect due to its low clindamycin resistance rate. The differentiation of virulence and resistance genes among subtypes provides a molecular basis for the precise prevention and control of MABC infection in tropical regions.

## Introduction

Non-tuberculous mycobacteria (NTM) are a group of environmental microorganisms widely present in water, soil and aerosols, which can invade the lungs, skin and bone marrow, etc. The most common is the invasion of the lungs, causing pulmonary infection (Wang et al., 2023). It is difficult to distinguish it from pulmonary tuberculosis based on clinical symptoms and imaging manifestations and other features, and it is very easy to be misdiagnosed and mistreated in clinical practice (Mwikuma et al., 2015). Among them, the *Mycobacterium abscessus* complex (MABC) serves as the representative of rapid-growing mycobacteria (RGM) (Medjahed et al., 2010). The clinical impact of MABC infection is significant and it has become one of the main pathogens causing refractory pulmonary infections worldwide. MABC is mainly divided into three subspecies: *M. abscessus* subsp. *abscessus* (Mab), *M. abscessus* subsp. *massiliense* (Mma), and *M. abscessus* subsp. *bolletii* (Mbo) (Wang et al., 2025). In recent years, with the global aging population, the rising prevalence of underlying lung diseases (such as COPD and bronchiectasis), and the widespread use of immunosuppressive agents, the incidence of MABC infections has been increasing year by year in both developed and developing countries. In East Asia, it has surpassed *Mycobacterium avium* complex (MAC) to become the most common pathogenic NTM (Nagano et al., 2017; Lim et al., 2018; Huang et al., 2017). The high pathogenicity of MABC is closely related to its unique biological characteristics. This bacterium has natural resistance to common disinfectants such as chlorine-based disinfectants (Victoria et al., 2021), organic mercury, and glutaraldehyde, and can form biofilms in hospital water systems and establish long-term colonization (Soverina et al., 2025). Its cell wall is rich in mycolic acid and glycolipids, which not only enhances its resistance to host immune clearance but also makes it difficult for traditional anti-tuberculosis drugs to penetrate. Additionally, the subspecies classification of MABC (Mab, Mma, Mbo) is closely related to its clinical phenotype and treatment response – for instance, Mma shows significantly higher sensitivity to macrolide drugs than other subspecies, with a cure rate of up to 80 to 90% (Choi et al., 2017), while the inducible drug resistance rate of Mab often exceeds 80% (Prommi et al., 2025; Wang et al., 2023). Although species identification is crucial for treatment decisions, traditional phenotypic methods (such as citrate utilization tests) have insufficient accuracy. Methods based on single-gene sequencing of *rpoB* or *hsp65* also have limitations due to the minor sequence differences among subspecies.

The development of whole-genome sequencing (WGS) technology has provided a new perspective for MABC research. Through single nucleotide polymorphism (SNP) analysis and Average Nucleotide Identity (ANI) calculation, WGS not only enables high-precision subspecies identification, but also reveals the transmission relationships and genetic evolution characteristics among strains (Gross et al., 2019). Recent studies have found that MABC-resistant strains may spread through interpersonal transmission, especially in hospital environments. This finding challenges the traditional belief that “NTM only infects through environmental exposure.” However, most existing research has focused on patients with cystic fibrosis (CF) (Bryant et al., 2013; Davidson et al., 2021). CF is more common among Caucasians, but it is extremely rare in some parts of Asia, including China (Li et al., 2017; Jin et al., 2022). The genomic characteristics of MABC in non-CF patients have not yet been clarified. Hainan, as the only tropical island province in China, has a hot and humid climate that may accelerate the reproduction of MABC in the environment. However, its molecular epidemiological characteristics, transmission routes, and resistance mechanisms remain unknown.

This study conducted WGS analysis on 113 MABC strains isolated clinically from tropical islands in China from 2014 to 2023 for the first time. The aim was to clarify the distribution of MABC subspecies in this region and the genetic correlations among the strains, to analyze the distribution characteristics of virulence genes and drug resistance genes, and to provide a theoretical basis for precise clinical diagnosis and treatment.

## Materials and methods

### Study subjects

Source of specimens: Respiratory specimens (such as sputum, bronchoalveolar lavage fluid, etc.) from permanent residents of Hainan who visited the Second Affiliated Hospital of Hainan Medical University (the only designated hospital for diagnosing and treating drug-resistant tuberculosis in Hainan) from January 2014 to June 2023 were collected for mycobacterium isolation and culture, as well as analysis of bacterial distribution and drug resistance characteristics.

Standard strains: The standard sensitive strain of *Mycobacterium tuberculosis* H37Rv, the standard strain of exotic Mycobacterium ATCC700686 (provided by the Tuberculosis Prevention and Control Clinical Center of the Chinese Center for Disease Control and Prevention); *Staphylococcus aureus* ATCC29213 (provided by the Clinical Laboratory Center of the Chinese Ministry of Health).

Inclusion criteria: On the premise of ensuring that the specimens are free from exogenous contamination, at least one strain of MABC was isolated and cultured from the patient specimens. When the same patient undergoes multiple cultures of the same bacterial strain, only the first strain during the study period is recorded.

Exclusion criteria: Patients with incomplete clinical data; Non-residents of Hainan area; Bacterial strains with failed quality control in WGS sequencing.

### Experimental methods

#### Isolation and screening of NTM

Mycobacterium isolation and culture: After the specimen is pre-treated, 0.15 mL (3 drops) is taken with a sterile pipette and inoculated onto the acidic Löwenstein-Jensen medium slant. The slant is placed upward and incubated for 24 h. Then it is inverted and further cultured.

NTM screening: Take fresh colonies and place them in the grinding culture bottle. Vortex for 0.5 to 1 min, then let it stand for 10 min. Take the supernatant and dilute it with sterile saline to be consistent with the MacFarland standard turbidity tube No.1 (1 mg/mL). Then dilute it to 10^−2^ mg/mL. Take 0.01 mL of the bacterial solution and streak it onto the PNB and TCH identification culture media. Cultivate at 37 °C. Observe for the first time on the third day, and then observe once a week for 4 weeks.

#### Bacterial strain identification and analysis

Initial identification: Take multiple colonies and transfer them into a nucleic acid extraction tube containing 80 μL nucleic acid extraction solution and magnetic beads. Shake for 10 min, then incubate at 95 °C in a water bath for 5 min, centrifuge at 12000 rpm for 1 min, and take 2 μL of the nucleic acid extract and add 18 μL of the PCR reaction system. After amplification, perform denaturation at 95 °C for 5 min and rapid cooling in ice bath for 3 min. Take out the PCR product, prepare the hybridization mixture, and add 13.5 μL of the hybridization mixture to the chip array for chip hybridization (at 50 °C for 2 h), then spin dry in the chip washing machine. Perform chip scanning and result interpretation.

Genetic sequencing identification: PCR amplification was performed for *hsp65* (*hsp65*-TB11: ACCAACGATGGTGTGTCCAT; *hsp65*-TB12: CTTGTCGAACCGCATACCCT, with 440 base pairs) and *rpoB* (Myco-F: GGCAGGTCACCCCGAAGGG; Myco-R: AGCGGCTGCTGGGTGATCATC, with 764 base pairs). After electrophoresis to verify the products, the effective PCR products were sent to Beijing Ruibo Xingke Biotechnology Co., Ltd. for bidirectional genetic sequencing. The bidirectional genetic sequences were spliced using DNAMAN (9) software, and the obtained gene sequences were subjected to gene comparison analysis on BLAST^1^.

#### *In vitro* drug susceptibility test of MABC strain

Preparation of the drug susceptibility plate: Using the microbroth dilution method, the drug susceptibility plate contains 12 types of antibacterial drugs: clarithromycin (CLR), amikacin (AK), moxifloxacin (MFX), linezolid (LZD), cefoxitin (FOX), tobramycin (TOB), amoxicillin/clavulanic acid (AMC), doxycycline (DOX), ciprofloxacin (CIP), imipenem (IPM), telithromycin (TGC), and trimethoprim/sulfamethoxazole (TMP-SMX).

Bacterial suspension dilution and inoculation: Take 2–5 mg of bacterial cells and transfer them to a grinding tube containing glass beads. Vortex and then dilute to a 0.5 McFarland concentration. Take 60 μL and add it to 12 mL of the inoculation solution. Add 100 μL of the diluted bacterial suspension to each well. Seal and incubate.

Culture: Incubate at 30 °C ± 2 °C for 72 h. If growth is poor, the incubation time can be extended to 48 h; then continue the culture for 14 days to detect macrolide-induced drug resistance.

Result interpretation: According to the CLSI critical concentration criteria (Clinical and Laboratory Standards Institute [CLSI], 2018) resistance (R), intermediate sensitivity (I), and sensitivity (S) are determined; MIC50 and MIC90 are calculated, and the smaller the values, the stronger the antibacterial activity.

#### Whole genome sequencing of MABC strains

Using a disposable sterile inoculation loop, a certain amount of bacteria was scraped and placed in 500 μL TE buffer. The MABC strain was then inactivated at 80 °C for 30 min. The inactivated MABC strain was immediately taken out and frozen at −80 °C in the refrigerator. It was transported with dry ice to Guangdong TB Healthcare Biotechnology Co., Ltd. for high-throughput sequencing and bioinformatics analysis to obtain the complete genome sequence.

##### Genome extraction, library construction and sequencing

Four hundred microliter of inactivated MABC bacterial strain culture was subjected to ultrasonic dispersion. DNA was extracted using the modified cetyltrimethylammonium bromide-lysozyme (CTAB) method (Larsen et al., 2007). The purity and concentration of the extracted DNA were determined using a Thermo NanoDrop 2000 micro-photometer and a Qubit Flurometer 3.0.

After quantification and quality inspection of the genomic DNA, the DNA was fragmented into nucleic acid fragments of 200–300 bp in length by a Covaris ultrasonic disruptor. The 5′ overhangs of the fragmented DNA were filled in or the 3′ overhangs were blunted. Adapters were ligated to both ends of the DNA fragments by T4 DNA ligase. The fragments were selected and purified by magnetic bead purification. Enrichment was achieved through PCR amplification. The reaction system was purified again by magnetic bead purification to remove primer dimers and other interferences, thereby obtaining a DNA library suitable for sequencing.

The concentration and length distribution of the libraries were determined using the Qubit Flurometer 3.0 fluorometer and Agilent 2,100, respectively. The qualified libraries were then run on the Illumina Novaseq 6,000 sequencing platform with a paired-end sequencing protocol (PE150).

##### Data quality control

The raw data (read sequences) were quality-controlled using the Fastp (0.20.0) quality control software. The raw data were also trimmed and filtered using the Trimmomatic (0.39) software (Bolger et al., 2014). The specific screening criteria are as follows: (1) Remove read segments containing adapters; (2) Eliminate read segments with a quality value (Q30) lower than 90%; (3) Remove read segments with a proportion of ambiguous bases (‘N’) exceeding 5%; (4) Trim low-quality bases at the ends of the read segments (quality threshold < 20). The cleaned read segments obtained through these criteria were used for subsequent analysis.

##### Bioinformatics analysis

The Shovill (1.1.0) (Rahmani et al., 2023) software was used to concatenate the clean reads to obtain the genomic Contigs for each sample. Quast (5.0.2) (Mikheenko et al., 2018) was employed to calculate the number of contigs, genome length, N50, and the maximum contig length of the concatenated genomes. Meanwhile, checkM2 (1.1.0) (Chklovski et al., 2023) was used to evaluate the completeness and contamination of the concatenated genomes (see Appendix S1). The annotated genomes were processed using Prokka (1.14.6) (Cuccuru et al., 2014) and the corresponding genome annotation files (.gff) were output. Kraken2 (2.1.3) (Wood and Salzberg, 2014; Boccacino et al., 2025) software was used to perform species annotation for the samples based on the NCBI species database.

Mutation analysis and phylogenetic tree construction: Based on the results of kraken2 (2.1.3) identification, the corresponding reference genome and its annotations were downloaded from NCBI. Based on the assembled genome contigs, mutation detection and annotation were completed using snippy (4.6.0). The core SNP sequences were output by the “snippy-core” command of snippy, and the SNP difference matrix of the entire genome was calculated and output using snp-dists (0.8.2). The phylogenetic tree was constructed using FastTree (2.1.10) by the maximum likelihood method based on the comparison of core SNP sequences. The phylogenetic tree was visualized and modified in iTOL (version 6.6)^2^ (Letunic and Bork, 2021).

Pan-genome analysis: Import the amino acid sequences (.faa files) from the annotation results of Prokka (1.14.6) into the BPGA (1.3) software for pan-genome analysis and draw the fitting curve of the pan/core genome openness model. Compare the variable genes (accessory), core genes (core), and specific genes (unique) to the KEGG database and calculate the proportion of genes in each pathway.

Molecular genotyping and genomic clustering: Compare the assembled genomic contigs to be tested with the reference genome sequences of the three subspecies of MABC, and calculate the average nucleotide identity (ANI) score of the entire genome using OrthoANI^3^ (Yoon et al., 2017). Genomes were identified as one subspecies based on an ANI of at least 98% (Li et al., 2024). The reference genomes were downloaded from NCBI: *M. abscessus* subsp. *abscessus* (Strain: ATCC 19977, ASM1718943v1), *M. abscessus* subsp. *massiliense* (Strain: JCM 15300, ASM49726v2), and *M. abscessus* subsp. *bolletii* (Strain: BD, ASM360971v1). Genotyping was performed using the PubMLST database^4^.

Analysis of virulence genes: Based on the virulence factor database (VFDB)^5^ database, the virulence genes related to the MABC strain were analyzed online. A heat map of virulence gene distribution was drawn using the Majorbio Cloud Platform (Han et al., 2024).

Analysis of drug resistance genes: Based on the online RGI main tool of The Comprehensive Antibiotic Resistance Database (CARD)^6^, the relevant drug resistance genes of the MABC strain were analyzed. The parameter settings were: Select Criteria: Only perfect and strict hits; Nudge: Loose hits to strict, Exclude nudge.

#### Statistical analysis

Statistical analysis was conducted using SPSS 25.0: For measurement data with normal distribution, *t*-test was used; for non-normal distribution, median and Mann–Whitney test were employed; for categorical data, chi-square test or Fisher’s exact probability method was applied. A difference was considered statistically significant when *p* < 0.05.

#### Data availability statement

The raw sequence data have been deposited in the Genome Sequence Archive at the National Genomics Data Center, China National Center for Bioinformation and Beijing Institute of Genomics, Chinese Academy of Sciences, under accession number CRA029244. These data are publicly accessible at https://ngdc.cncb.ac.cn/gsa.

## Results

### Demographic data of patients

**Figure 1:**
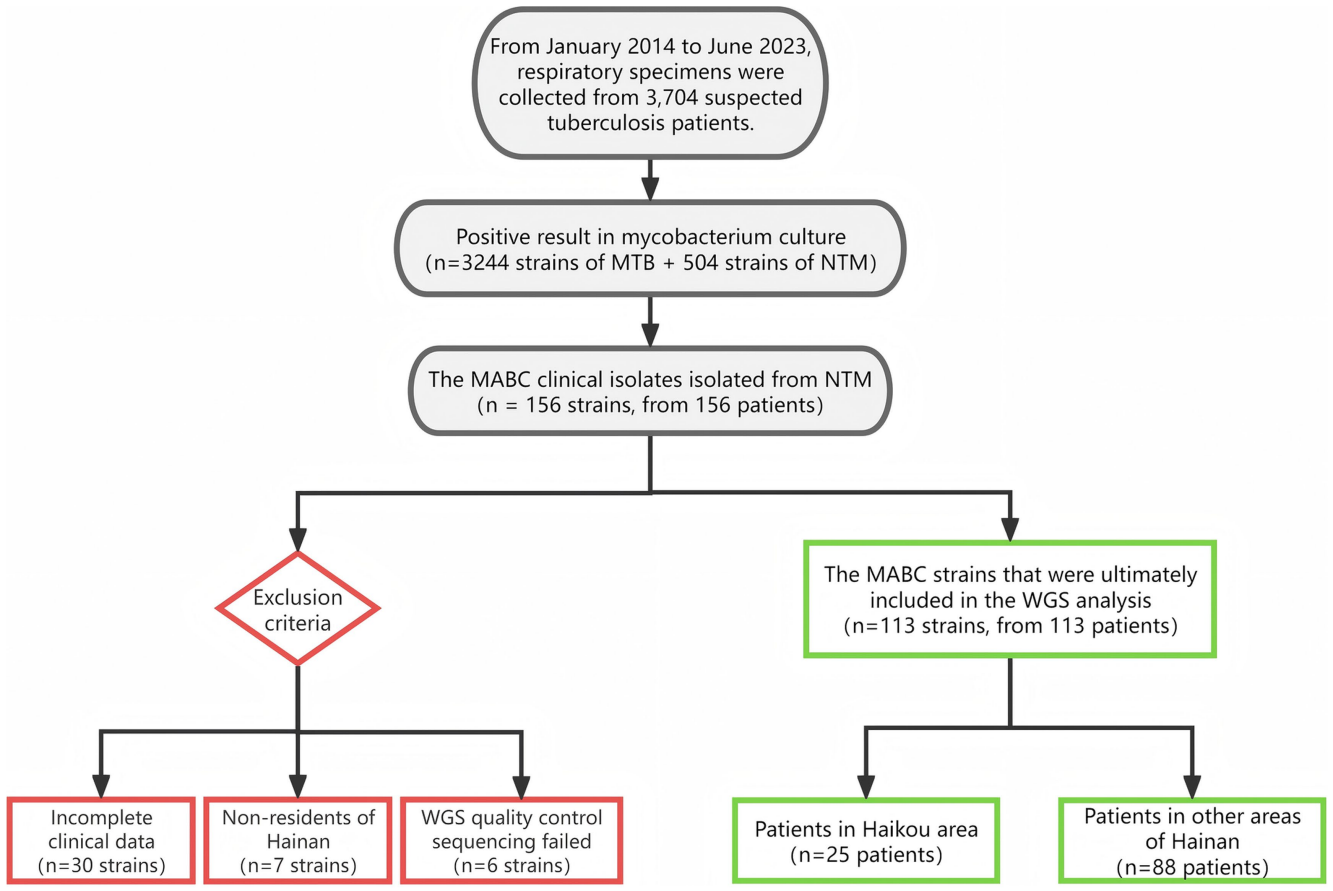
Flowchart of MABC strain screening and inclusion process.

From January 1, 2014 to June 30, 2023, 3,244 strains of *Mycobacterium tuberculosis* (MTB) and 504 strains of NTM were isolated from the respiratory specimens of 3,704 suspected tuberculosis patients. Among the 156 patients, clinical isolates of MABC were isolated. After excluding 30 patients with incomplete clinical data, 7 patients from other provinces, and 6 isolates with substandard sequencing quality control or genomic contamination, a total of 113 isolates (72.4%, 113/156) of MABC bacteria from 113 patients were included in the WGS analysis. Among them, 25 isolates were from Haikou (22.1%, 25/113) (as shown in Figure 1).

The median age of 113 patients with MABC infection was 64.0 years (54.0, 72.0). Among them, 42 were male, with an age of 65.0 years (51.0, 79.2); 71 were female, with an age of 64.0 years (56.0, 68.0). The male-to-female ratio was 0.59:1.

### Genotyping identification and genomic clustering of MABC molecules

The molecular genotyping of the complete genome sequences of 113 MABC strains using ANI identified that Mab was the most common (65.5%, 74/113), followed by Mma (33.6%, 38/113), and Mbo was the least common (0.9%, 1/113).

**Figure 2:**
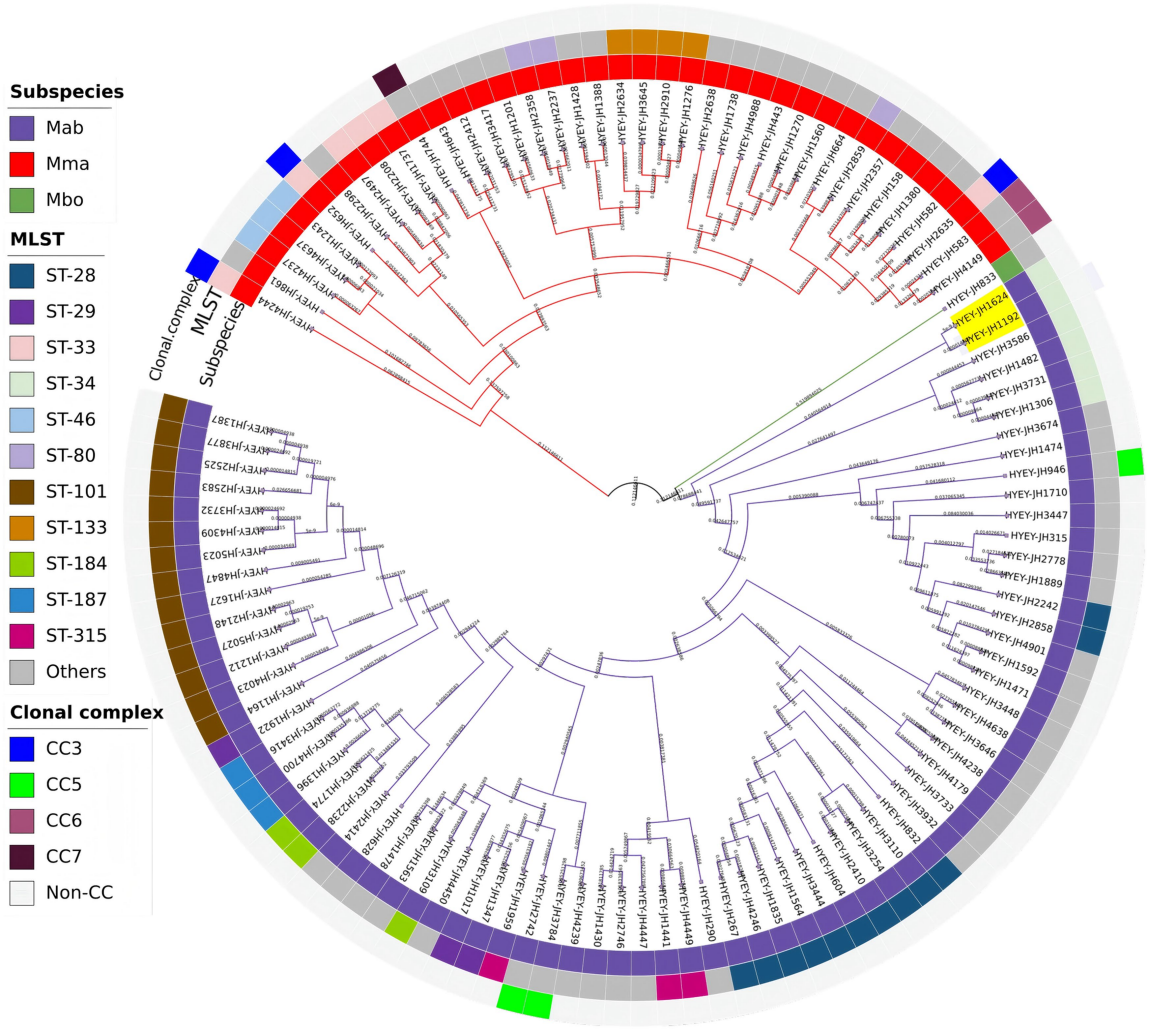
A phylogenetic tree was constructed based on the core SNP sequences of 113 MABC strains. The genetic relationship between the strains is presented using the maximum likelihood method. The branch colors represent different subspecies. The node labels are the strain numbers, and the branch lengths represent the genetic distance. The yellow annotations in the figure indicate a pair of strains that differ by only 5 SNPs (HYEY-JH1192 and HYEY-JH1624).

Based on the analysis of whole-genome SNPs, this study identified two pairs of Mab strains with extremely close genetic distances (Supplementary Table S4). Strain HYEY-JH1192 (isolated in August 2018) and strain HYEY-JH1624 (isolated in June 2019) differ by only 5 SNPs, and this difference value is lower than the threshold of ≤7 SNPs typically used to determine possible recent transmission (Wang et al., 2025; Davidson et al., 2022). In addition, strain HYEY-JH1387 (isolated in January 2019) and strain HYEY-JH3877 (isolated in January 2022) differ by 8 SNPs and their genetic distances are also highly similar. The phylogenetic analysis (Figure 2) further confirmed the close relationship between these two Mab strains. Each of them forms a highly supported independent terminal clade branch on the evolutionary tree, suggesting that each pair of strains has a recent common ancestor. The pairwise SNP differences among all the other strains are significantly greater than the above values, indicating a highly diverse overall genetic background.

The MLST analysis based on the PubMLST database revealed that among the 113 MABC strains, there were 53 different sequence types (STs). Among them, 4 STs have more than 5 members (14st-101, 12st-28 and 6st-34 are all Mab strains, and 6st-33 are all Mma strains), while the other STs have fewer than 5 strains each, and 31 STs each consist of only 1 strain. Among the 74 Mab strains, there are 34 types of STs, and among the 38 Mma strains, there are 18 types of STs. Only 1 Mbo strain is ST-110. At the same time, 4 previously reported Clonal complexes (CC) were further identified (Jin et al., 2022) (CC3, CC5, CC6, and CC7, from the PubMLST database), CC3 was formed by 3 Mma ST-33 strains, CC6 by 2 Mma ST-39 strains, CC7 by 1 Mma ST-42 strain, and CC5 was formed by 1 Mab ST-23 strain and 2 Mab ST-99 strains (as shown in Figure 2).

### MABC pan-genome analysis

#### Pan-genome structural characteristics

The pan-genome analysis of 113 MABC strains from Hainan Province revealed (Supplementary Table S5): The core genome is highly conserved. All 113 strains contain 3,626 core gene families, accounting for 79.8% of the average genome size. These mainly involve basic metabolic, DNA replication, and transcription and translation functions (Tettelin et al., 2005).

The number of accessory genes showed significant variation (883 ± 113 genes per strain), indicating that there are a large number of non-essential functional genes among the strains.

Unique genes are widely distributed across strains. Notably, several strains, including HYEY-JH861 (212 genes), HYEY-JH2497 (211 genes) and HYEY-JH582 (184 genes), possess a significantly expanded set of unique genes. The biological functions and evolutionary origins of these unique genes remain to be further elucidated.

The exclusively absent genes were significantly enriched in some strains. For instance, strain number HYEY-JH1270 (Mma) lacked 51 genes. These deletions may be related to ecological adaptation or the evolution of drug resistance.

**Figure 3:**
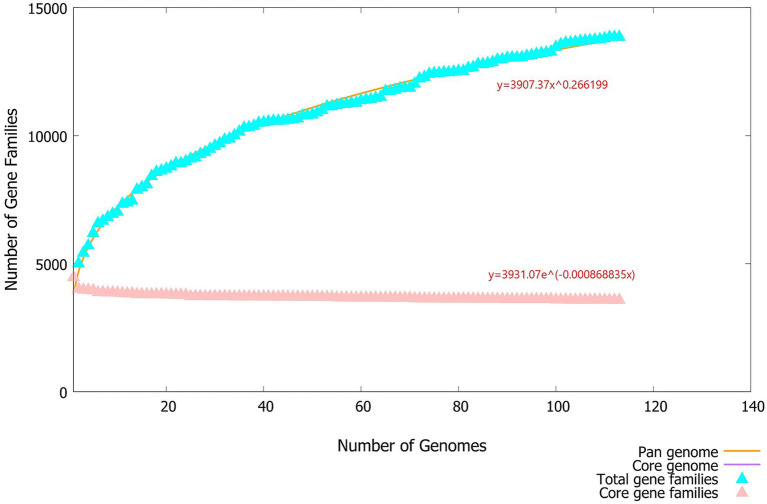
Fitting curve of the pan-genome of 113 MABC strains. The pan-genome fitting curve shows the trend of changes in the size of the MABC pan-genome and the core genome as the number of sequenced strains increases. The fitting parameter *b* = 0.266199 indicates that the pan-genome is open-ended, meaning that new genes continue to increase as new strains are added and have not reached a plateau.

The pan genome exhibits an open structure (the parameter ‘b’ = 0.266199) (Li et al., 2024; Vernikos et al., 2015) (Figure 3), indicating that as new genes are introduced, the pan genome continues to expand. It expands continuously with the increase in the number of strains (not reaching the plateau stage), but has not reached saturation, suggesting that this population has significant genetic diversity (Tettelin et al., 2008).

#### Trend of new gene addition

**Figure 4:**
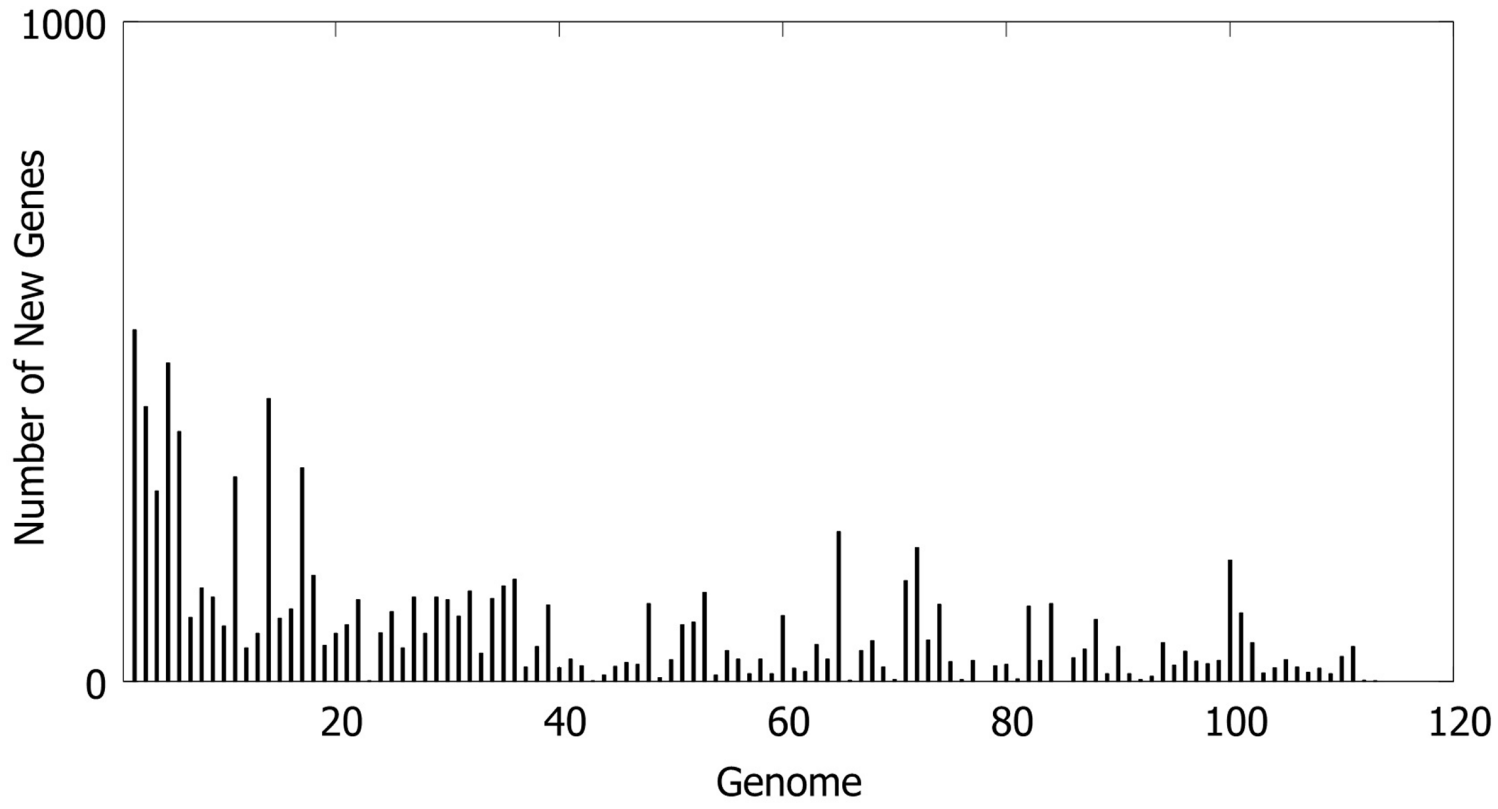
Distribution of new gene quantities. The horizontal axis represents the number of strains, and the vertical axis represents the cumulative number of new genes.

As the number of sequenced strains increased, the discovery rate of new genes (New genes) was the highest in the early stage of the analysis (<20 strains), and continued to increase up to 113 strains (Figure 4). This indicates that there are a large number of rare genes in the Hainan MABC population, supporting the open nature of its pan-genome.

#### Distribution of function categories

**Figure 5:**
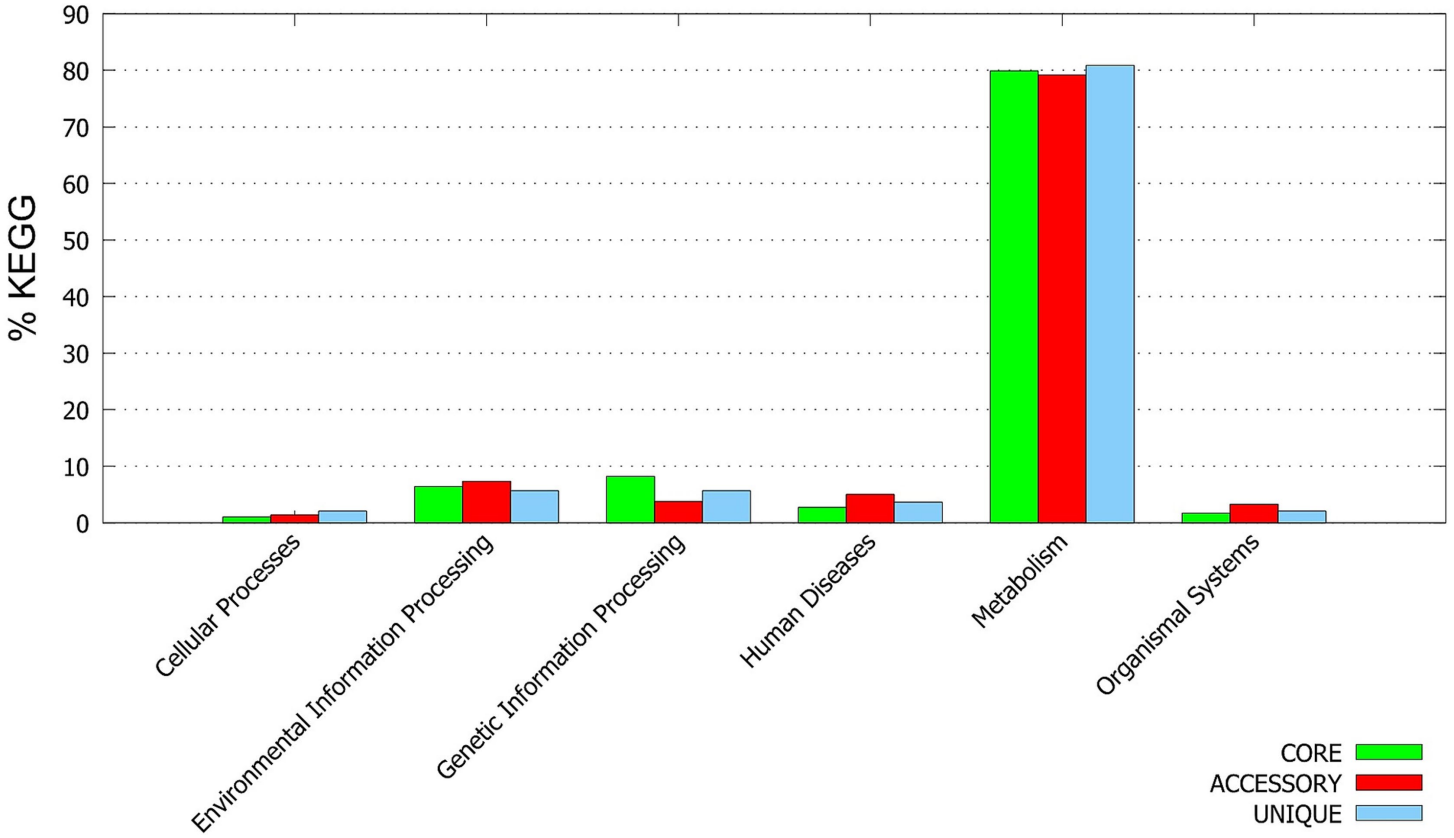
KEGG functional annotation. Based on the KEGG database, the functions of the core genes, accessory genes and unique genes of MABC were classified and statistically analyzed. The bar chart shows the distribution ratios of various genes in pathways such as “Metabolism,” “Genetic Information Processing,” “Environmental Information Processing,” “Cell Processes,” and “Human Diseases”.

KEGG functional annotation shows (Figure 5): The core genes are mainly enriched in the “Metabolism” and “Genetic Information Processing” categories; the accessory and unique genes have a higher proportion in the pathways related to “Environmental Information Processing” and “Human Diseases,” suggesting that these genes may play a role in environmental adaptation and pathogenic mechanisms.

#### Genomic divergence among subspecies

The Mab strain had a higher number of accessory genes than the Mma strain (*p* = 0.005, Mann–Whitney test), but there was no significant difference in unique genes (*p* > 0.05, Mann–Whitney test), which might be related to the species-specific ecological adaptability.

The Mma strain with the number HYEY-JH4244 has 177 unique genes and 17 specific missing genes, suggesting that it may have undergone co-evolution of gene acquisition and loss in a specific ecological niche.

### Analysis of virulence genes of MABC

**Figure 6:**
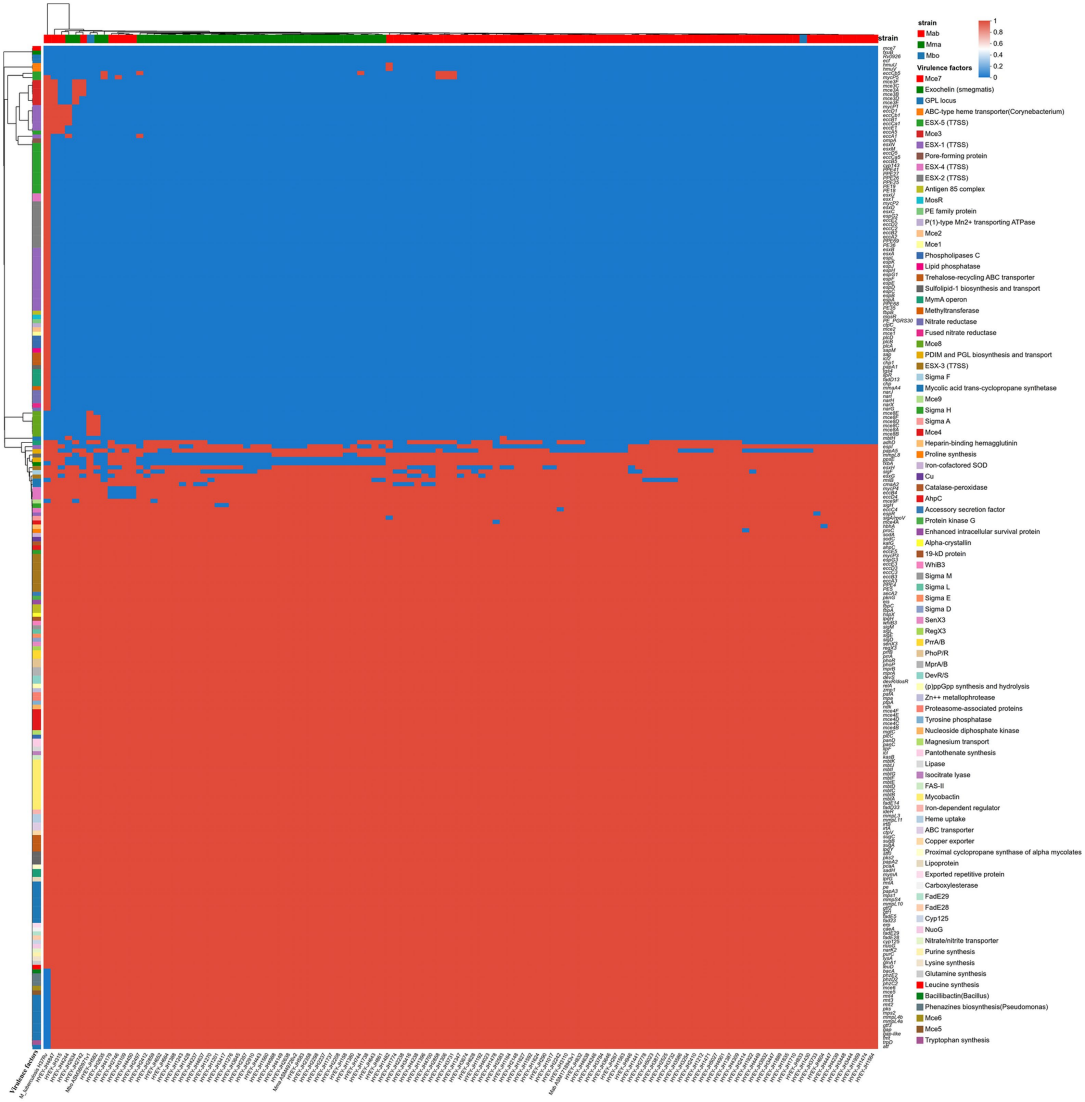
Heatmap of virulence gene distribution of 113 MABC strains. The heatmap shows the presence (in red) and absence (in blue) of the 113 MABC virulence genes. The rows represent the bacterial strains, and the columns represent the virulence genes.

A systematic analysis of the distribution of virulence genes in 113 MABC strains was conducted, and a total of 138 to 158 virulence-related genes were identified (as shown in Figure 6). These genes encode various factors required for colonization in the host, including adhesion, cell penetration, and avoidance of immune clearance. The core findings are as follows (Supplementary Table S6):

#### Highly conservative virulence mechanism

97.9% (234/239) of the virulence factors showed no significant differences among the subspecies (*p* > 0.05), including:

Basal metabolism pathway: Amino acid synthesis (*gluA1*, *leuD*, *lysA*, etc.) and purine metabolism (*purC*) are present in all strains (100.0%).

Catabolism of cholesterol: *cyp125*, *fadE28*, *fadE29* 100.0% conserved (*p* > 0.05);

Iron uptake system: The entire gene cluster for mycolate synthesis (*mbtA-mbtK*) and the iron regulatory factor *ideR* are conserved in all strains.

ESX secretion system: The core components of ESX-3 (*eccA3*, *eccB3*, *eccC3*, *eccD3*, *eccE3*) are all present at 100%, and the presence rate of key genes of ESX-4 (*eccB4*, *eccC4*, *eccD4*) is over 95.0%.

Stress defense: Catalase *katG* (100.0%), Superoxide Dismutase *sodA*, *sodC* (100.0%).

#### Significant virulence factors

Through chi-square test/Fisher’s exact test, 5 types of significantly different virulence factors were identified (*p* < 0.05) (Table 1).

**Table 1:** The significantly different virulence genes among the MABC subspecies.

Toxicity category	Gene	The prevalence of Mab (n/n)	The prevalence of Mma (n/n)	*X* ^2^	*p*-value	Mab ASM1718943v1	Mma ASM49726v2	Mbo ASM360971v1	MTB H37Rv
PDIM and PGL biosynthesis and transport	*papA5*	47.3% (35/74)	92.1% (35/38)	21.508	<0.001	−	+	−	+
PDIM and PGL biosynthesis and transport	*ppsE*	98.6% (73/74)	0.0% (0/38)	107.654	<0.001	+	−	+	+
Sulfolipid-1 biosynthesis and transport	*mmpL8*	100.0% (74/74)	39.5% (15/38)	56.364	<0.001	+	+	+	+
Exochelin synthase	*fxbA*	98.6% (73/74)	0.0% (0/38)	107.653	<0.001	+	−	+	−
Regulatory factor	*sigF*	89.2% (66/74)	65.8% (25/38)	9.024	0.003	+	+	+	+

The number of Mbo is relatively small and is not included in the differential analysis; “+” indicates the presence of the gene; “–” indicates the absence of the gene.

GPL, Glycopeptidolipid; PDIM, phthiocerol dimycocerosate; PGL, phenolic glycolipid.

### Results of MABC drug sensitivity test

**Figure 7:**
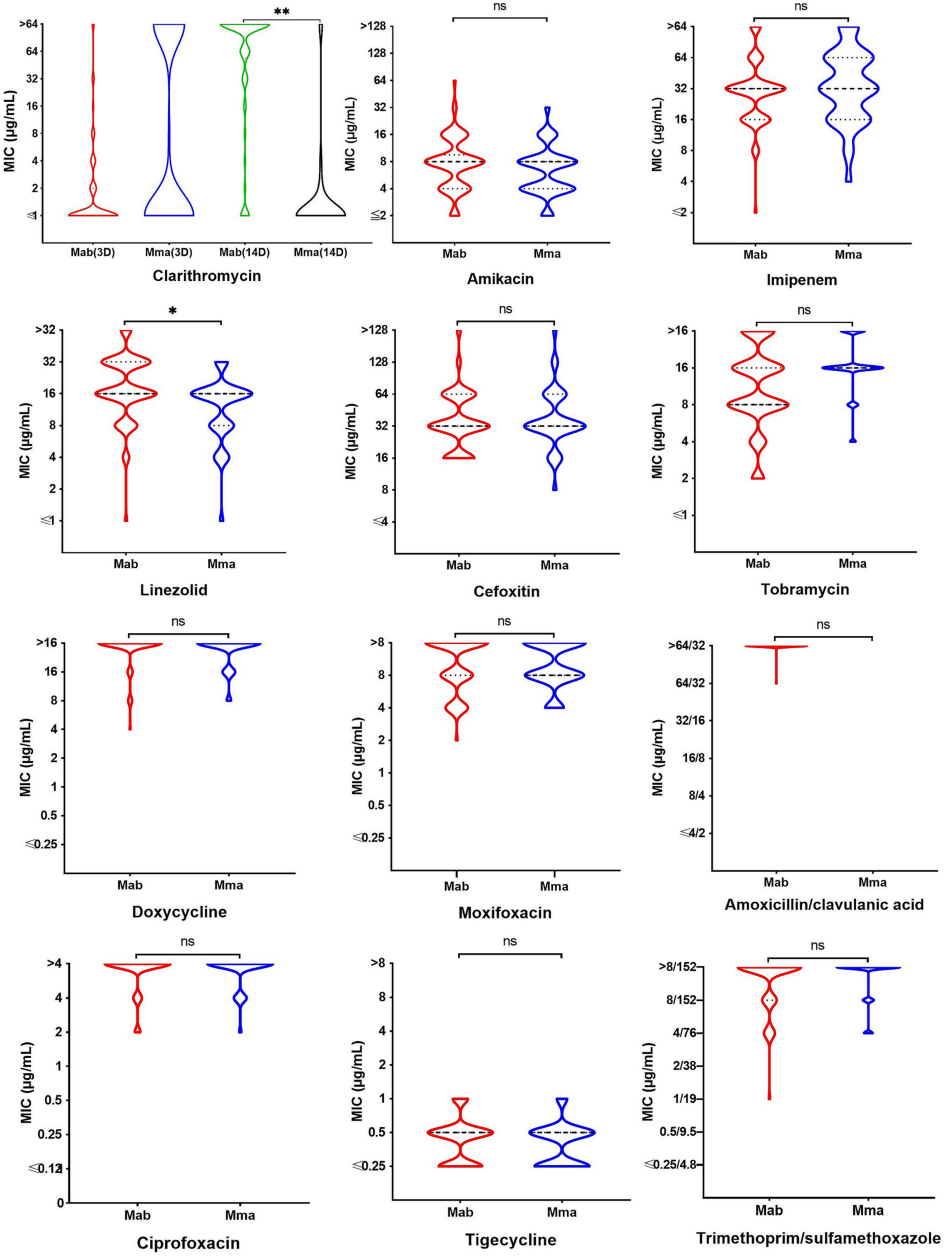
Comparison of the differences in MIC distribution of drugs for Mab and Mma strains. ns represents *p* > 0.05, *represents *p* < 0.05, **represents *p* < 0.001.

The resistance rates of the Mab and Mma strains to DOX, MFX, AMC, CIP and TMP-SMX all exceeded 90%. The resistance rate induced by CLR (14D) in Mab (86.5%) was the highest, while that in Mma was only 5.3%, showing a statistically significant difference (*p* < 0.001). Additionally, there was also a statistically significant difference in the resistance rate to LZD between Mab and Mma (*p* < 0.05) (Figure 7 and Table 2).

**Table 2:** The sensitivity of MABC to antimicrobial agents was determined by MIC method.

Drug	Broth dilution ranges (μg/mL)	Drug allergy breakthrough*			Mab (*N* = 74)					Mma (*N* = 38)				
		S (μg/mL)	I (μg/mL)	R (μg/mL)	MIC50	MIC90	S (%)	I (%)	R (%)	MIC50	MIC90	S (%)	I (%)	R (%)
Amikacin (AK)	2 ~ 128	≤16	32	≥64	8	16	95.9%	2.7%	1.4%	8	16	97.4%	2.6%	0.0%
Clarithromycin (CLR) (3D)	1 ~ 64	≤2	4	≥8	≦1	8	82.4%	6.8%	10.8%	≦1	≦1	97.4%	2.6%	0.0%
Clarithromycin (CLR) (14D)	1 ~ 64	≤2	4	≥8	>64	>64	12.2%	1.4%	86.5%	≦1	2	92.1%	2.6%	5.3%
Imipenem (IPM)	2 ~ 64	≤4	8 ~ 16	≥32	32	64	1.4%	29.7%	68.9%	32	>64	2.6%	28.9%	68.4%
Linezolid (LZD)	1 ~ 32	≤8	16	≥32	16	32	20.3%	40.5%	39.2%	16	32	36.8%	52.6%	10.5%
Cefoxitin (FOX)	4 ~ 128	≤16	32 ~ 64	≥128	32	64	23.0%	71.6%	5.4%	32	64	15.8%	76.3%	7.9%
Tobramycin (TOB)	1 ~ 16	≤2	4	≥8	8	>16	6.8%	9.5%	83.8%	16	>16	0.0%	5.3%	94.7%
Doxycycline (DOX)	0.25 ~ 16	≤1	2 ~ 4	≥8	>16	>16	0.0%	1.4%	98.6%	>16	>16	0.0%	0.0%	100.0%
Moxifloxacin (MFX)	0.25 ~ 8	≤1	2	≥4	>8	>8	0.0%	1.4%	98.6%	8	>8	0.0%	0.0%	100.0%
Amoxicillin/clavulanic acid (AMC)	4/2 ~ 64/32	≤8/4	16/8	≥32/16	>64/32	>64/32	0.0%	0.0%	100.0%	>64/32	>64/32	0.0%	0.0%	100.0%
Ciprofloxacin (CIP)	0.12 ~ 4	≤1	2	≥4	>4	>4	0.0%	6.8%	93.2%	>4	>4	0.0%	2.6%	97.4%
Tigecycline (TGC)**	0.25 ~ 8	≤4	–	>4	0.5	1	100.0%	-	0.0%	0.5	0.5	100.0%	–	0.0%
Trimethoprim/sulfamethoxazole (TMP-SMX)	0.25/4.8 ~ 8/152	≤2/38	–	≥4/76	>8/152	>8/152	1.4%	-	98.6%	>8/152	>8/152	0.0%	–	100.0%

*CLSI M24, 3rd ed. 2018 (2).

**CLSI recommended that only MIC values be reported for Tigecycline, and this interpretation was set as a reference point for clinical reference.

MIC50: 50% minimum inhibitory concentration.

MIC90:90% minimum inhibitory concentration.

### Analysis of MABC resistance genes

#### Widespread distribution of core drug resistance genes

All 113 MABC strains (including Mab, Mma and Mbo) carried multiple drug resistance genes (Table 3):

**Table 3:** Antibiotic resistance genes found among Chinese MABC isolates.

ARO Term	Mab (*n* = 74)	Mma (*n* = 38)	Mbo (*n* = 1)	Mutation	Drug	Drug resistance mechanism
MAB	74	38	1	/	Cephalosporin, penicillin beta-lactam	Antibiotic inactivation
RbpA	74	38	1	/	Rifamycin antibiotic	Antibiotic target protection
*vanY* gene in *vanB* cluster	74	38	1	/	Glycopeptide antibiotic	Antibiotic target alteration
*rrs*	73	38	1	a1408g	Aminoglycoside antibiotic	Antibiotic target alteration
*rrl*	72	38	1	a2059g	Macrolide antibiotic	Antibiotic target alteration
*qacJ*	0	37	0	/	Disinfecting agents and antiseptics	Antibiotic efflux
Erm(41)	3	0	0	/	Macrolide antibiotic, lincosamide antibiotic, streptogramin antibiotic, streptogramin A antibiotic, streptogramin B antibiotic	Antibiotic target alteration

MAB Class A *β*-lactamase was present in 100% of the strains (113/113), and it mediated the inactivation of *β*-lactam antibiotics (such as penicillin, cephalosporins). Among them, 25 strains (22.1%) were “Perfect” matches (100% sequence consistency), suggesting a high level of resistance.

RbpA (RNA polymerase binding protein) was found in 100% of the strains (113/113), conferring Rifamycin resistance by protecting the antibiotic target.

The *vanY* glycopeptide resistance gene cluster was present in 100% of the strains (113/113), mediating the modification of the target sites of glycopeptide antibiotics such as vancomycin, but the sequence consistency was relatively low (27.1–31.5%), suggesting potential functional variations.

#### Key drug resistance mediated by ribosomal RNA mutations

The *rrs* a1408g mutation (mediating amikacin resistance) was present in 99.1% of the strains (112/113), and was associated with resistance to amikacin and other aminoglycoside drugs. The sequence identity was as high as 99.5% (“Curated-R” level, indicating that the drug resistance has been verified by the database).

The *rrl* a2059g mutation (which mediates macrolide resistance) was present in 98.2% of the strains (111/113), and was directly associated with clarithromycin resistance. The sequence identity was ≥99.8%.

#### Species-specific drug resistance characteristics

The Mma gene was significantly enriched for the qacJ efflux pump gene (97.4%, 37/38), mediating the efflux of disinfectants/antibiotics.

Three Mab strains and one Mbo strain carry the Erm(41) methyltransferase gene (with sequence identity > 98.5%), which can lead to cross-resistance to macrolides, lincosamides and chloramphenicol B.

## Discussion

This study conducted a systematic analysis of 113 clinical isolates of *Mycobacterium abscessus* complex (MABC) from tropical islands in China (Hainan) using whole-genome sequencing (WGS) technology. It was the first to reveal the genomic characteristics, subspecies differentiation, and drug resistance mechanisms of MABC in this region, providing a new perspective for the precise prevention and control of NTM infections in tropical areas. The following discussion is carried out from three dimensions: genomic epidemiological characteristics, adaptive evolution of virulence genes, and clinical implications of drug resistance mechanisms.

### Genomic epidemiological characteristics and infection routes

The MABC strain in Hainan has Mab (65.5%) and Mma (33.6%) as the predominant prevalent strains, while Mbo accounts for an extremely low proportion (0.9%). This is different from the studies conducted in Beijing (Hou et al., 2025) and Shanghai (Guo et al., 2020; Li et al., 2024) in temperate regions, suggesting that the tropical high-temperature and high-humidity environment may affect the population structure of MABC.

This study, through whole-genome SNP analysis, identified a pair of Mab strains (HYEY-JH1192 and HYEY-JH1624) that differed by only 5 SNPs in two patients (with a time interval of 10 months between the isolation of these two strains). This difference value was below the common threshold for molecular epidemiological transmission (≤ 7 SNPs) (Wang et al., 2025; Davidson et al., 2022). This constitutes preliminary molecular evidence of possible interpersonal transmission or a common source outbreak. This is a key finding of this study, suggesting that in certain circumstances, MABC may have a local transmission risk rather than being completely independently acquired from the environment. At the same time, the discovery of another pair of Mab strains differing by 8 SNPs (the HYEY-JH1387 and HYEY-JH3877 strains, with an isolation time interval of up to 3 years) is also worthy of attention. Although slightly above the aforementioned strict threshold and not supporting a typical recent transmission event, such a high degree of genetic similarity strongly suggests that they originated from a long-standing common environmental reservoir or a chronic infection source. The patient may independently contract the infection over a long period of time by coming into contact with the same continuously contaminated environment (such as the hospital water supply system) (Victoria et al., 2021). The discovery of these two pairs of strains, especially the 5-SNP pair, has changed our initial assessment of the transmission risk of MABC in this area, suggesting that in the predominant mode of environmental exposure, local potential common exposure risks still need to be paid attention to. Therefore, we need to re-evaluate and strengthen infection control measures, and conduct a retrospective thorough investigation of the epidemiological associations (such as temporal and spatial intersections, common medical exposure histories) of the relevant patients. If the epidemiological investigation can confirm the association, it will provide important evidence for the intrahospital transmission of MABC.

Pangenome analysis further revealed the significant genetic diversity of the MABC population in Hainan region. The analysis indicated that the core genome of this population was highly conserved, containing 3,626 core gene families, accounting for approximately 79.8% of the average genome size, and mainly encoding proteins necessary for basic metabolism and housekeeping functions (Tettelin et al., 2005; Cummins et al., 2022).

In sharp contrast to this, the accessory genomes and unique genes exhibit a wide range of variations. For instance, some strains (such as HYEY-JH861, HYEY-JH2497) carry over 200 unique genes. These unique genes show an unstable strain-specific distribution, suggesting that they may have been acquired through horizontal gene transfer events (such as mediated by plasmids or bacteriophages) from external sources. It is particularly noteworthy that such genes may be captured in the specific ecological niches of hospitals, enabling the strains to acquire adaptive genes related to disinfectant tolerance, antibiotic resistance, etc. (Davidson et al., 2022). This hypothesis awaits systematic verification in the future through the application of specialized bioinformatics tools such as plasmid prediction and phage identification.

Further analysis confirmed that this pan-genome exhibited a distinct open feature (the parameter ‘b’ = 0.266199), indicating that as the number of sequenced strains increased, new genes would continuously be incorporated into the gene pool. This open genetic background enabled it to rapidly acquire exogenous genes (including resistance and virulence genes) through horizontal gene transfer, thereby significantly enhancing its environmental adaptability (Tettelin et al., 2005). Particularly importantly, recent studies have confirmed that MABC can acquire resistance plasmids and trigger clonal spread in hospital environments through this mechanism (Davidson et al., 2021). This suggests that the openness of the pan-genome provides a genetic basis for the continuous evolution and spread of multi-drug resistant bacterial strains, posing a significant challenge to clinical infection control.

The functional annotation (KEGG) analysis of the accessory and unique genes revealed that these genes were significantly enriched in pathways related to environmental signal response, stress resistance, and host interaction (Figure 5). This might suggest that these variable gene pools could play a crucial role in adapting to the unique tropical climate and complex ecological environment (including natural environment and hospital microenvironment) of Hainan Province. For instance, some unique genes were annotated as drug efflux pumps or known virulence factors, which might be directly related to the survival and competitive advantage of the strains in specific habitats.

### Subspecies-specific differentiation of virulence genes

The core virulence mechanism of MABC is highly conserved among different strains, including cholesterol metabolism pathways (such as *cyp125*, *fadE28*, etc.), iron uptake system (*mbtA-mbtK* gene cluster), and ESX-3 secretion system, ensuring its survival ability within host macrophages (Jin et al., 2022; Griffin et al., 2012; Serafini et al., 2013; Dubois et al., 2018). However, the key virulence genes show species-specific differentiation, which may be directly related to the differences in clinical phenotypes:

Differential expression of lipid metabolism genes: The detection rate of phenolic glycolipid synthesis gene *papA5* in Mma (92.1%) was significantly higher than that in Mab (47.3%), possibly by synthesizing phenolic glycolipids to inhibit the host’s TNF-*α* signaling pathway (Jin et al., 2022) and enhance immune evasion; Mab highly expressed the synthesis gene *ppsE* of PDIM (98.6%), and it is speculated that it promotes the formation of biofilms by strengthening the hydrophobicity of the cell wall (Converse et al., 2003), which is related to the preference for skin infections. The high expression of *mmpL8* in Mab (100.0% vs. 39.5% in Mma) may alter the surface charge of the bacteria by transporting *sulfatide-1*, thereby reducing the adhesion of host antimicrobial peptides (Dubois et al., 2018). This mechanism is consistent with the role of *sulfite-1* in *Mycobacterium tuberculosis* in inhibiting the maturation of phagosomes (Domenech et al., 2004). However, the specific function of sulfite in MABC remains unclear and requires further experimental verification.

Function compensation of the secretion system: MABC lacks the ESX-1 system of *Mycobacterium tuberculosis* (which mediates the escape from phagosomes); the ESX-3 core gene is intact in both subspecies and the standard strain, consistent with MTB H37Rv, indicating that its function of chelating host iron ions through secretion of iron carriers (such as mycolic acid) is non-replaceable (Siegrist et al., 2014).

Subspecies differences in stress response genes: The detection rate of *σ* factor sigF in Mab (89.2%) was significantly higher than that in Mma (65.8%). As a stress regulatory factor, *sigF* can enhance the dormancy ability of bacteria in hypoxic and nutrient-deficient environments, and may be related to the persistent colonization of Mab in chronic infections. Genes such as *katG* and *sodA* are conserved in all strains, supporting that MABC maintains cell survival by decomposing host reactive oxygen species (ROS) (Bates et al., 2024).

### Drug resistance mechanism and clinical treatment implications

The drug sensitivity test and the analysis of drug resistance genomes revealed the multi-drug resistance characteristics and species-specific drug resistance phenotypes of MABC, providing crucial evidence for clinical treatment:

Universal presence of inherent resistance genes: All strains carry the MAB *β*-lactamase (which hydrolyzes *β*-lactam drugs), RbpA (rifampicin resistance), and the *vanY* glycopeptide resistance gene cluster, consistent with the high resistance phenotype to β-lactams and glycopeptides.

Ribosome mutations lead to high-level resistance: 99.1% of the strains have the *rrs* a1408g mutation (for amikacin resistance), and 98.2% carry the *rrl* a2059g mutation (for macrolide resistance), directly resulting in the clinical failure risk of first-line drugs such as amikacin and clarithromycin (Li et al., 2024).

Subspecies-specific drug resistance phenotype: The 14-day induced resistance rate of Mma to clarithromycin (5.3%) was significantly lower than that of Mab (86.5%), and this result is consistent with the conclusion of a multi-center study in China (Liu et al., 2021).

It is worth noting that in this study, there is a complex relationship between the drug-resistant genotypes and phenotypes. Although a wide range of drug-resistant genes were annotated through the CARD database, overall, some classic mutations were highly consistent with the phenotypes. However, we also found significant inconsistencies. The most prominent one was the absence of a necessary connection between the presence of the Erm(41) gene and the clarithromycin-induced drug resistance phenotype. Previous studies suggested that the high induction resistance rate of Mab and Mbo to clarithromycin might be related to the Erm(41)T28 genotype (Bastian et al., 2011; Richard et al., 2020). However, this study found that clarithromycin-induced resistance was associated with a “decoupling” phenomenon of the Erm(41) gene-only 3 of the Mab bacteria carried a fully functional Erm(41) gene. This “decoupling” phenomenon strongly suggests that in the MABC strains prevalent in this region, there may exist a new clarithromycin-induced resistance dominant mechanism that is not dependent on Erm(41), such as 23S rRNA epigenetic modification (a2059g mutation) or drug efflux mediated by efflux pumps (Victoria et al., 2021). This finding explains why the genotypic prediction based on the Erm(41) gene in this study cohort did not match the phenotype, emphasizing the necessity of conducting clarithromycin-induced susceptibility tests for Mab and Mbo in clinical practice, which cannot be replaced by genetic testing. Other resistance genes, such as the widely present MAB *β*-lactamase gene, are consistent with a high-level resistance phenotype to β-lactam drugs, while the enrichment of the *qacJ* efflux pump gene in Mma is related to its potential disinfectant tolerance adaptation in the hospital environment, suggesting the need to strengthen microbial monitoring of hospital water systems.

This study has the following limitations: (1) the samples for this study were all collected from the only designated hospital for drug-resistant tuberculosis in Hainan Province. Although the MABC strains included in the study covered the entire Hainan region and could reflect the main clinical strain characteristics of the region, there was still a regional concentration. In the future, it is necessary to combine multi-center and environmental samples to comprehensively reveal the full epidemiological picture of MABC in Hainan; only 1 strains of the Mbo subtype were included, restricting comparisons between subtypes; (2) insufficient verification of transmission routes: Although genetic analysis indicated that environmental exposure was the main infection route, and two pairs of genetically highly similar strains were identified, there was a lack of direct genomic comparisons of environmental samples (such as hospital water sources, soil), and thus the empirical closure of the transmission chain could not be completed. (3) The exploration of the drug resistance mechanism is not thorough: Regarding the phenomenon of clindamycin-induced resistance “decoupling” from the Erm(41) gene, only the existence of alternative mechanisms (such as efflux pumps or epigenetic modifications) was hypothesized, but the specific molecular pathways were not verified through experiments, and the functional activities of vanY and other resistance genes lacked phenotypic correlation analysis; (4) This study did not include MABC strains from other geographical regions in China (such as the temperate regions) or from Southeast Asia for comparative genomic analysis. Such comparisons would help clarify whether the genetic diversity and subspecies distribution observed in Hainan are unique to this tropical environment or part of a broader regional trend. Future studies that can integrate public genomic data from different geographical environments and conduct systematic comparisons will be crucial for accurately defining the regional representativeness of this finding and for revealing the adaptive evolution driven by climate and environmental factors. (5) Lack of systematic analysis of mobile genetic elements: This study did not use specific tools to predict and annotate the plasmids and prophages of all strains. Therefore, the discussion in the article regarding the possible association of unique genes with horizontal gene transfer is mainly based on the speculation of gene distribution patterns, and requires subsequent studies to provide direct evidence of genomic structure.

## Conclusion

This study has for the first time systematically revealed the genomic characteristics and drug resistance mechanisms of MABC in tropical islands of China (Hainan). The clinical isolates from Hainan MABC mainly belong to the Mab and Mma subtypes, and show a high degree of genetic diversity in total. It is notable that the whole-genome SNP analysis identified two pairs of strains with highly similar genetics (with differences of 5 and 8 SNPs respectively), and the difference value of one pair was lower than the commonly used transmission threshold, suggesting that there are molecular clues of local common exposure or potential transmission events. This finding is worthy of attention and needs to be clarified in future studies by combining epidemiological investigations. Overall, the research results support that environmental exposure is the main route of infection in this region. The drug sensitivity test showed that the induction of resistance to clarithromycin by Mma was significantly lower than that by Mab, indicating a better treatment outcome. Analysis of virulence genes reveals that the differentiation of lipid metabolism and secretion systems between subspecies may affect host adaptability, while the open nature of the pan-genome suggests that this population enhances its environmental adaptability by acquiring new genes. The widespread distribution of core drug resistance genes (such as MAB *β*-lactamase, *rrs/rrl* mutations) is closely related to the high-level drug resistance phenotype. Some strains carrying acquired resistance genes (such as *qacJ*) indicate a potential risk of horizontal gene transfer. This study provides important scientific basis for the molecular epidemiology research and precise diagnosis of MABC infection in the Hainan region. Future work should increase the sample size and focus on combining in-depth epidemiological investigations and environmental tracing to clarify the transmission routes. At the same time, through functional experiments, the molecular mechanisms of key genes should be analyzed to provide theoretical basis for the prevention and control of MABC infection in tropical islands.

## Data Availability

The datasets presented in this study can be found in online repositories. The names of the repository/repositories and accession number(s) can be found in the article/Supplementary material.

## Footnotes

1
https://blast.ncbi.nlm.nih.gov/Blast.cgi

2
https://itol.embl.de/

3
https://www.ezbiocloud.net/tools/ani

4
https://pubmlst.org/bigsdb?db=pubmlst_rmlst_seqdef

5
https://www.mgc.ac.cn/cgi-bin/VFs/v5/main.cgi

6
https://card.mcmaster.ca/analyze/rgi
